# St.Gallen consensus on safe implementation of transanal total mesorectal excision

**DOI:** 10.1007/s00464-017-5990-2

**Published:** 2017-12-12

**Authors:** Michel Adamina, Nicolas C. Buchs, Marta Penna, Roel Hompes, on behalf of the St.Gallen Colorectal Consensus Expert Group, Michel Adamina, Felix Aigner, Matthew Albert, Stephen Bell, Willem Bemelman, Luigi Boni, Carl J. Brown, Gina Brown, Nicolas C. Buchs, Felix Grieder, Ulrich Güller, Roel Hompes, André d’Hoore, Cristiano Huscher, Masaaki Ito, Werner Kneist, Joep Knol, Antonio Lacy, Justin Maykel, Arend Merrie, Jae Hwan Oh, Yves Panis, Marta Penna, Rodrigo Oliva Perez, Frank Pfeffer, Philip Quirke, Philippe Rouanet, Eric Rullier, Gerald Seitinger, Colin Sietses, Antonino Spinelli, Andrew R. L. Stevenson, Patricia Sylla, Paris Tekkis, Jean-Jacques Tuech, Jurriaan Tuynman, Janindra Warusavitarne, Mark Whiteford, Des Winter, Albert Wolthuis

**Affiliations:** 1 Department of Surgery, Cantonal Hospital Winterthur, Brauerstrasse 15, Postfach 834, 8401 Winterthur, Switzerland; 2 University of Basel, Basel, Switzerland; 3 Department of Surgery, University Hospital Geneva, Geneva, Switzerland; 4 Department of Colorectal Surgery, University Hospitals of Oxford, Oxford, UK; 5 Department of Surgery, Academic Medical Centre, Amsterdam, The Netherlands

**Keywords:** Rectal cancer, Transanal total mesorectal excision, Guidelines, Oncology

## Abstract

**Background:**

The management of rectal cancer has evolved over the years, including the recent rise of Transanal Total Mesorectal Excision (TaTME). TaTME addresses the limitations created by the bony confines of the pelvis, bulky tumours, and fatty mesorectum, particularly for low rectal cancers. However, guidance is required to ensure safe implementation and to avoid the pitfalls and potential major morbidity encountered by the early adopters of TaTME. We report a broad international consensus statement, which provides a basis for optimal clinical practice.

**Methods:**

Forty international experts were invited to participate based on clinical and academic achievements. The consensus statements were developed using Delphi methodology incorporating three successive rounds. Consensus was defined as agreement by 80% or more of the experts.

**Results:**

A total of 37 colorectal surgeons from 20 countries and 5 continents (Europe, Asia, North and South America, Australasia) contributed to the consensus. Participation to the iterative Delphi rounds was 100%. An expert radiologist, pathologist, and medical oncologist provided recommendations to maximize relevance to current practice. Consensus was obtained on all seven different chapters: patient selection and surgical indication, perioperative management, patient positioning and operating room set up, surgical technique, devices and instruments, pelvic anatomy, TaTME training, and outcomes analysis.

**Conclusions:**

This multidisciplinary consensus statement achieved more than 80% approval and can thus be graded as strong recommendation, yet acknowledging the current lack of high level evidence. It provides the best possible guidance for safe implementation and practice of Transanal Total Mesorectal Excision.

The management of rectal cancer has evolved over the years with several options available to physicians taking care of cancer patients, including refined neoadjuvant and adjuvant therapies and various surgical techniques. Among the newly developed surgical approaches to rectal cancer, transanal total mesorectal excision (TaTME) proposes to address the anatomical limitations of the bony confines of the pelvis, bulky tumours, and fatty mesorectum through a new approach. Indeed, while taking advantage of the magnification of a laparoscope, performing a total mesorectal excision through the anus may confer a number of benefits. In particular a different viewpoint with a facilitated excision of the lower third of the mesorectum, better visualization of the endangered structures during dissection, and potentially a safer anastomosis by avoiding the multiple stapler firings too often required in a conventional complete anterior approach. Following the first live case in 2009 and inspiring experience of the pioneers [1, 2], dissemination of TaTME is taking place swiftly with many institutions having adopted this technique and published encouraging results. A large, international registry documents the adoption and practice of TaTME, with more than 2500 procedures from 39 different countries and 128 active centres recorded so far [3]. Furthermore, two international randomised controlled trials from the COLOR [4] and GRECCAR [5] investigators have recently started, randomising patients between TaTME and conventional laparoscopic TME. COLOR III [4] and GRECCAR 11 [5] are expected to help define the place and true value of TaTME in the surgical armamentarium for mid and low rectal cancer. Meanwhile, guidance is required to ensure safe implementation of TaTME, avoiding the pitfalls and intra-operative complications encountered and overcome by the pioneers and early adopters of this promising technique. The objectives of this international and interdisciplinary consensus statement are three-fold:

1. to provide a framework and guidance to those embarking on TaTME, including patient selection and surgical indication, technique, and educational opportunities;
2. to highlight the challenges, benefits, and distinctive dangers of this technique, capitalizing on a large international experience of early adopters of TaTME;
3. to promote prospective outcomes analysis and participation into clinical trials and registries.

Hence, it is hoped that the present international consensus guidelines will provide a basis for optimal clinical practice.

## Materials and methods

### Sponsor and potential conflict of interest

The consensus was sponsored by the European Colorectal Congress of St.Gallen, Switzerland (http://www.colorectalsurgery.eu), which covered all costs associated with the entire consensus process with no support or involvement from the medical industry. The European Colorectal Congress is organizing one of the three largest colorectal congresses worldwide with an attendance of over 1400 participants from 80 countries (2016). It has no corporate sponsor, no member of the medical industry on its Board, and it does not own stock or participation to any medical company.

The four core authors of the consensus ensured scientific integrity. They completed the International Committee of Medical Journal Editors (http://www.icmje.org) form for disclosure of potential conflict of interest, reporting nothing to disclose. They were not paid for their time and efforts. The single benefit granted to the 40 consensus experts beyond collaborative authorship was not having to pay to attend the European Colorectal Congress in 2016.

Importantly, all statements referring to devices and instruments used to perform TaTME have been generated by the four core authors, in agreement with the current literature and routine practice of most experts. Referring to a given product and its alternatives does not imply endorsement of any manufacturer, it does solely provide the technical guidance based on the experience of a large group of international experts that may be expected by readers of the consensus.

### Consensus development

The consensus process was based on current recommendations for guideline developments adapted to the question at hand [6]. The core authors of the consensus drafted its agenda and formulated the initial questions with a focus on current practice, areas of controversy, and educational perspective. They identified and invited a group of international experts based on their clinical and academic achievements in the field of rectal cancer surgery and TaTME. Experts had performed at least 20 TaTME cases and reported their results in peer-reviewed publications and registries. They were major contributors to the international Low Rectal Cancer taTME registry (http://www.lorec.nhs.uk) having reported together more than 1000 TaTME procedures. An expert pathologist, radiologist, and medical oncologist were invited to participate to ensure the highest standard of care from a multidisciplinary team in the recommendations of the consensus.

The consensus statements were developed using a Delphi methodology [7] incorporating three successive rounds. The first two consecutive rounds were web-based with anonymous voting, and explicitly asked for feedback and suggestions from the international experts. The comments recorded were included into the iterative development of the consensus statements. The third round was a dedicated expert meeting during the European Colorectal Congress in St.Gallen on November 30th, 2016 with face-to-face open discussion and finalisation of the consensus document. Consensus was defined as agreement by 80% or more of the experts. The final manuscript was then drafted by the four convenors of the consensus with only minor editing of the consensus statements if required. The discussion further developed practical advice, including perspectives from the expert radiologist and oncologist. The final consensus document was reviewed and approved by all involved experts.

Regarding the paucity of clinical data published on TaTME, no formal grading of evidence was provided. The authors of most major cohort series on TaTME were experts of the present consensus. Recommendation strength of the consensus statements was graded according to the Grading of Recommendations, Assessment, Development and Evaluation system (GRADE) [6]. Unresolved controversies and discussion points complete each section of the consensus.

An update of the present consensus and systematic review of the literature is planned within three years by the core authors.

## Results

Forty international experts were invited to participate in the consensus process. Two experts delegated participation to another senior staff surgeon who fulfilled the expertise criteria defined. A total of 37 colorectal surgeons from 20 countries in 5 continents (Europe, Asia, North and South America, Australasia) contributed to the work. The percentage reported in the consensus statements refers to those 37 (100%) expert colorectal surgeons. Participation to the first 2 web-based Delphi rounds was 100%, while 30 of 37 (81.1%) colorectal surgeons attended the third round live. The remaining 7 experts who could not attend the third round in person, televoted on the revised statements, achieving 100% participation to the third round. The consensus panel included three additional experts from the fields of clinical histopathology, radiology, and medical oncology. All 40 experts approved the final version of the manuscript.

### Consensus statements

#### Patient selection and surgical indications

Both genders can be operated on by a transanal approach. The female pelvis tends to be broader and therefore allows for an easier mesorectal excision. Obesity, especially visceral obesity with a fatty mesorectum, is an important limitation. Lastly, bulky mid/distal rectal tumours are very challenging, in both female and male patients. Hence, a TaTME may be technically easier than an abdominal TME in patients with a narrow pelvis, obese patients, and patients bearing a bulky mid/distal rectal tumour.

37/37 = 100%

In males, the prostatic urethra is at risk during the dissection of the lower third of the rectum. Injury to the prostatic urethra, especially when the pelvis has been irradiated, is a major complication.

37/37 = 100%

In females, the vagina is at risk of injury during the dissection of the lower third of the rectum. This lesion can be directly repaired with simple sutures, even in an irradiated pelvis. Caution must be taken when fashioning the anastomosis to avoid the dreaded complication of a neorecto-vaginal fistula.

37/37 = 100%

Prior pelvic surgeries make any total mesorectal excision more difficult, irrespective of the abdominal or transanal approach. Operating on patients with a prior prostatectomy, especially for an anterior rectal cancer with close circumferential resection margin (CRM), can be challenging. Prior mesh rectopexy may also pose greater surgical challenges. A prior hysterectomy is usually not a limitation.

34/37 = 91.9%

No further gender limitation/preference were felt relevant.

37/37 = 100%

There are no given body mass index (BMI) or limitation in BMI which make TaTME much better than open/laparoscopic/robotic TME.

37/37 = 100%

There is no given hip-waist ratio which makes TaTME much better than open/laparoscopic/robotic TME.

37/37 = 100%

### Rectal cancer height

Transanal total mesorectal excision is best for lower rectal resections. It can be performed for partial mesorectal excision, e.g. for a cancer of the upper third of the rectum, although the need for an endoscopic purse-string placement on the long rectal stump increases the technical challenges. Caution should be taken not to perform unnecessary total mesorectal excision. The minimum distal margin of 1 cm applies to lower third cancers, whereas upper and middle third cancers require a distal margin of 5 cm. A partial mesorectal excision can be safely performed for cancer of the upper third of the rectum, whereas a total mesorectal excision is required for cancer of the mid and lower third.

32/37 = 86.5%

When an abdominoperineal excision is indicated, a “transperineal TaTME approach” may be undertaken once the surgeon has sufficient expertise in TaTME.

32/37 = 86.5%

Potential benefits of a “transperineal TaTME approach” include tailoring the dissection to the oncologic needs and avoiding the need to flip the patient in prone position in order to obtain an accurate view of the anterior plane.

34/37 = 91.9%

An intersphincteric TaTME can also be performed, thereby preserving some of the sphincter function. The known benefits of TaTME then apply, particularly better visualization of the lower two-thirds of the rectum when dissecting upwards. An intersphincteric TaTME requires, however, a coloanal or colo-pouch-anal handsewn anastomosis and the corresponding surgical expertise. Furthermore, the anterior dissection can be very difficult in an intersphincteric resection placing the urethra at risk.

37/37 = 100%

### Surgical indications beyond rectal cancer

Beyond the classical surgical indication for neoplastic disease, TaTME can be performed in the context of inflammatory bowel disease. A proctectomy alone or a proctocolectomy can be performed, with or without ileal pouch anal anastomosis. For benign diseases, especially when a pouch reconstruction is considered, dissection of the rectum close to the bowel wall is an option that offers better function.

36/37 = 97.3%

Pouch advancement procedures, the dissection/removal of a neorectum in cases of chronic anastomotic sinus/anastomotic leak, and proctectomy for rectovaginal fistula are advanced procedures which can be performed transanally if appropriate technical and surgical expertise is available. The underlying disease, local inflammation, and dissection through scar tissue and obscured planes may be challenging.

37/37 = 100%

### Perioperative management

#### Enhanced recovery

The recommendations for the perioperative management of TaTME patients follow the principles of enhanced recovery pathways (ERAS). The ERAS society has published in 2013 its guidelines for perioperative care in elective rectal/pelvic surgery [8], which the present group of experts endorses and recommends for the safe practice of TaTME.

36/37 = 97.3%

### Mechanical bowel preparation

Full mechanical bowel preparation in all patients in whom a total mesorectal excision is planned is recommended, irrespective of the use of a diverting ostomy [9, 10].

33/37 = 89.2%

### Pelvic drain

Evidence is scarce to use a routine pelvic drain after TaTME. Its use depends mainly on the surgeon’s preference.

34/37 = 91.9%

### Urinary catheter

A urinary catheter can be removed safely on the first postoperative day with low catheter reinsertion rates, including in elderly males. Epidural analgesia does not prevent early catheter removal. A suprapubic catheter is a good option whenever prolonged postoperative urinary drainage is anticipated.

30/37 = 81.1%

### Perioperative antibiotic prophylaxis

Perioperative antibiotic prophylaxis and its repetition during the operation are mandatory and follow institutional guidelines. There is no evidence to support an extended perioperative antibiotic prophylaxis beyond 24 h.

37/37 = 100%

### Patient positioning and operative room set-up

#### Patient preparation

The standard set-up may vary. However, a lithotomy position (or modified Lloyds-Davies position) is mandatory to allow a good position and exposition for the abdominal and perineal teams.

37/37 = 100%

Insertion of a urinary catheter, particularly in males, is advised. It may help to achieve a safer anterior dissection. In addition, by withdrawing the transanal platform, it allows palpation of the prostatic urethra in case of doubt.

34/37 = 91.9%

A generous rectal washout is advised after completing the pursestring and before starting the transanal dissection.

30/37 = 81.1%

#### One versus two-team approaches

A one or two-team procedure can be performed and both have their advantages and disadvantages [11]. The two-team approach is costlier, at least in terms of personnel (two surgical and scrub teams). However, it should save at least 30 min of operative time and in case of difficult dissection it allows a better visualization and the two operating surgeons can help each other.

33/37 = 89.2%

The consensus panel advises to operate with two teams simultaneously whenever possible. Yet, a single operating team switching between abdominal and transanal approaches can also be very effective.

37/37 = 100%

On the other hand, a two-team approach requires a good collaboration between the abdominal and perineal teams. An integrated operative theatre should be advised to assure a good view of the screens. Space around the patient may be, however, an issue when two complete operative teams work synchronously.

30/37 = 81.1%

For a one-team approach, the benefits of starting the TaTME abdominally include exclusion of a peritoneal carcinomatosis, early splenic flexure mobilization and vascular control, and easier identification of the left ureter and autonomic nerves at the promontory level. In addition, the risk of massive distension of the colon (due to insufficiency of the rectal purse-string) is reduced.

32/37 = 86.5%

In a one-team approach, initiating the pelvic dissection from above does not seem to limit the extent and quality of the pneumopelvis.

35/37 = 94.6%

When starting the TaTME transanally, a crucial point in the operation is swiftly securing an air-tight purse-string. This avoids stool contamination, cancer cell spillage, and bowel dilatation.

37/37 = 100%

Starting the TaTME transanally allows for an exact transection point of the rectum assuring correct assessment of the distal margin.

32/37 = 86.5%

### Devices and instruments

#### Scope

A 10 mm high definition scope is preferred as it offers a broader visual field. For the transanal dissection, a 30° scope is recommended. A 3D system allows superior depth visualization; however, it is routinely used only in a few centres as there is a lack of data on reported clinical benefits.

32/37 = 86.5%

For the abdominal part, a 5 mm or a 10 mm scope may be used. Ten millimetres scopes offer a broader and brighter view.

37/37 = 100%.

#### Transanal access platforms

A stable transanal access platform is required to ensure a pneumorectum and insertion of three ports. Most experts use a GelPOINT Path access platform (Applied Medical, Rancho Santa Margarita, CA, USA) inserted transanally [12]. Many alternative platforms from major suppliers exist (DalimSurgNet, Ethicon, Medtronic, Olympus, Storz, Wolff, etc).

34/37 = 91.9%

When performing an abdominoperineal excision, a transanal access platform is inserted when the dissection reaches the depth of the levator ani.

32/37 = 91.1%

Alternative platforms may be used depending on the surgeon’s preference. The expert panel has limited experience with alternative platforms, and hence specific recommendation on these cannot be made (e.g. some experts supported TEO Storz (59.5%, Tuttlingen, Germany), TEM Wolff (40.5%, Knittlingen, Germany), Octo-Port (21.6%, DalimSurgNet, Seoul, Korea), etc).

33/37 = 89.2%

Anal retraction sutures or an anal retractor system (most experts used a Lone Star retractor, CooperSurgical, Trumbull, CT, USA) may prove useful, especially when performing a handsewn anastomosis.

36/37 = 97.3%

#### Insufflation

Transanal CO_2_ insufflation should ensure a stable pneumorectum/pneumopelvis under continuous smoke evacuation, a much helpful feature as the transanal dissection occurs close to the scope [13]. Most experts used an Airseal system (CONMED, Utica, NY, USA) for this purpose.

The abdominal part of the procedure may use a standard insufflator. Alternatively, two standard air insufflators can be used concurrently for the transanal and the transabdominal stages. However, the transanal insufflator should be able to deliver high insufflation pressure to compensate for frequent to constant smoke evacuation.

33/37 = 89.2%

The pneumorectum is typically initiated through an access port using low pressure/low smoke evacuation levels. It is important to occlude the rectal lumen with an abdominal clamp until the pursestring is completed and the rectal dissection is started. Upon progression of the rectal dissection a higher CO_2_ pressure and smoke evacuation level are required, up to 20 mmHg. For a standard high insufflation pressure the recommended abdominal pressure is 12–15 mmHg.

32/37 = 86.5%

### Laparoscopic instruments

Standard laparoscopic instruments are used for the transanal dissection. Monopolar cautery is used most frequently; alternatively, an energy device can be used, although this may further increase the procedure cost.

31/37 = 83.8%

The panel selected monopolar and bipolar cautery as the preferred energy source for the transanal dissection. Of all other energy sources (bipolar sealing device, ultrasonic shears, or a combination thereof in a single instrument), ultrasonic shears were used by a minority (2/37 = 4.6%) for transanal dissection.

35/37 = 94.6%

Curved/angulated instruments may be useful.

30/37 = 81.8%

Transanal extraction of the specimen using a wound protector may be envisaged, depending on the size of the tumour and the bulkiness of the specimen. However, avoiding an abdominal extraction incision must be balanced against the risk of damage to both the sphincter complex and the specimen in a transanal extraction.

36/37 = 97.3%

When extracting the specimen through an abdominal incision, a wound protector should be used to prevent port site metastases and wound infection.

37/37 = 100%

### Pelvic anatomy revisited: the transanal perspective

Recognizing visual clues and orientating oneself are at the core of a safe surgical procedure [14]. The pelvic anatomy seen through the transanal perspective is novel even to experienced surgeons. Several pitfalls may arise from leaving the correct plane. Early recognition of errors and return to the correct plane are crucial to a safe TaTME dissection.

37/37 = 100%

In females, the anterior dissection carries the risk of entering the vagina. Usually, this injury can be easily recognized and repaired as required.

36/37 = 97.3%

The urethra in males may be injured during the initial anterior dissection.

35/37 = 94.6%

If in doubt, the transanal platform should be removed and the prostate/prostatic urethra/urinary catheter palpated to confirm the correct dissection plane.

34/37 = 91.9%

There is a risk of following a perimuscular plane and therefore being too close to the rectum and/or cancer. This is especially true at the beginning of the transanal dissection, when caution should be undertaken to identify and proceed early within the TME plane.

36/37 = 97.3%

Pneumopelvis may create areolar planes beyond the dissection point thus leading the surgeon astray.

36/37 = 97.3%

The ureters are particularly at risk of injury during the anterolateral dissection, especially when the lateral dissection is carried out too widely without control from an abdominal surgeon.

32/37 = 86.5%

Caution should be undertaken to avoid too lateral a dissection during the transanal approach, because of the risk of injury to the pelvic side wall and its structures.

37/37 = 100%

Posteriorly, too deep a plane runs the risk of entering the presacral space with possible subsequent injury to the presacral venous plexus.

36/37 = 97.3%

For low rectal tumours, an (total or partial) intersphincteric dissection may be required but carries the risk of poor function.

35/37 = 94.6%

### TaTME training

#### Courses, proctoring and mentoring

TaTME represents an important addition to the contemporary treatment of rectal pathologies. In particular, it has the potential to improve the outcomes in rectal cancer surgery. However, the safe and successful introduction and development of TaTME requires adequate training. Participation in dedicated courses, including hands-on/cadaveric courses, taking part in a mentoring/proctoring program, and performing initial TaTME cases under supervision are crucial steps in the safe learning and implementation of TaTME.

37/37 = 100%

The consensus panel advises to participate in a TaTME course prior to performing any TaTME cases in the clinical setting. A TaTME course should include peer-reviewed materials covering pelvic anatomy from the transanal perspective, surgical technique of TaTME, pitfalls, and technical troubleshooting.

37/37 = 100%

Furthermore, it is important for the whole multidisciplinary team to know the particulars of TaTME. Case observation and hospital visit, involving one’s complete theatre team, are very useful prior to starting one’s first TaTME.

37/37 = 100%

In addition to case observation, mentoring/proctoring with an expert surgeon available is strongly advised.

36/37 = 97.3%

An important further prerequisite is adequate experience in oncological rectal surgery, including an annual centre volume of at least 10 cases.

36/37 = 97.3%

The learning curve for safe and independent practice of TaTME is yet to be established but progress is slow even for the experienced laparoscopic colorectal surgeon. Depending on previous laparoscopic TME and TEM/TAMIS experience, 1–5 TaTME cases should be proctored/supervised before embarking on solo practice.

35/37 = 94.6%

The panel agrees that the overall learning curve is long and demanding, with more than 20 cases required. No consensus could be reached on a given number of procedures to reach proficiency.

37/37 = 100%

Prospective monitoring and benchmarking one’s own outcomes is advised, as well as participation in clinical studies. In particular, perioperative clinical outcomes, oncological outcomes, and function/quality of life should be monitored in an effort to continuously improve quality.

37/37 = 100%

#### Surgical technique step by step

The consensus panel endorses a standardized surgical technique to allow for safe and reproducible outcomes. Several publications have described ad hoc surgical techniques and variations in performing TaTME. In the context of an upcoming randomised controlled trial evaluating TaTME vs laparoscopic TME (COLOR III), a step by step TaTME procedure has been validated and published. This consensus panel recommends adherence to the surgical technique described in the COLOR III protocol [15].

34/37 = 91.9%

TaTME starts with either the transabdominal or transanal phase. In the abdominal phase the sigmoid and the splenic flexure are mobilised by multiport laparoscopy or through single port surgery with the single port located in the future ileostomy site. The inferior mesenteric artery is centrally ligated after identification of the left ureter. After mobilisation of the descending colon, sigmoid and the proximal rectum, the transanal phase is initiated.

In a two-team approach, the lumen of the distal sigmoid colon is occluded early with a grasper to minimize colonic distension while the perineal surgeon completes the pursestring.

31/37 = 83.8%

The transanal phase starts with a washout of the rectum with a povidone-iodine solution [16, 17]. Use of anal retraction sutures and/or an anal retractor are advised.

37/37 = 100%

For distal tumours (< 5 cm from the anal verge), an intersphincteric dissection is performed with the use of an anal retractor. The transanal dissection is continued as proximal as possible in open fashion. Thereafter, the open rectum is closed with purse-string suture to prevent spillage of bacteria and tumour cells.

33/37 = 89.2%

In case of tumours above 5 cm from the anal verge, a transanal platform is inserted and sutured to the perineal skin. The rectal stump is then closed with a pursestring suture with a recommended minimum distance of 1 cm from the distal end of the cancer. This pursestring suture can be placed through the transanal platform under direct vision or endoscopically, especially for more proximal tumours.

32/37 = 86.5%

A pneumorectum is created with carbon dioxide at a pressure of 14 mmHg and a relatively low flow of 5 l per minute to minimise rectal contractions. When dissection progresses, insufflation settings can be increased and air evacuation controlled to allow for the best possible visualization. Use of a dedicated insufflation management system, which provides a stable pneumopelvis under continuous smoke evacuation, is advised.

34/37 = 91.9%

Dissection starts by marking the distal resection level with the diathermy hook, then proceeding to a full thickness incision of the rectal wall. The dorsal plane is then developed proximally using blunt and cautery dissection along the TME plane. The ventral dissection comes next, taking great care not to injure the vagina and to preserve the prostatic urethra. The lateral dissection comes last after progression of the dorsal and ventral parts, in order to minimize the risk of damaging neurovascular structures. Lastly, the peritoneal reflection is opened. This step should be carried out last as it may markedly impair the ability to maintain a pneumopelvis.

30/37 = 81.1%

Whenever restoration of bowel continuity is envisaged, construction of a diversion ileostomy is advisable to protect against and minimize the risk of anastomotic leak [18, 19].

33/37 = 89.2%

There are different techniques to perform a low anastomosis after TaTME. If an intersphincteric dissection was performed, a handsewn coloanal anastomosis is the best option.

37/37 = 100%

On the other hand, if there is enough distal rectum to perform a pursestring, a stapled anastomosis can be safely performed.

35/37 = 94.6%

Different anastomotic techniques have been published [20] and seem feasible and safe. To date, there is no strong evidence supporting one particular technique. All stapled anastomoses use a double pursestring technique placed at the rectal stump and at the colonic end. Multiple stapler firings with crossing staple lines is thus avoided. The diameter of the stapler varies from 28 to 33 mm.

36/37 = 97.3%

The type of reconstruction depends on the surgeon’s preference and the patient’s anatomy (end-to-end or side-to-end anastomosis, or colonic J pouch). There is limited data suggesting better short-term functional outcomes with a side-to-end or colonic J pouch reconstruction.

35/37 = 94.6%

Depending on the size of the specimen, an abdominal or a transanal extraction can be performed. A transanal extraction reduces the risk of postoperative abdominal wall complications (pain, wound infection, incisional hernia). It may, however, put additional stretch on the anal sphincter and can cause trauma to the rectal specimen.

34/37 = 91.9%

However, there are cases where a transanal extraction is not possible or suitable (short mesentery, bulky specimen, risk of specimen injury). In this situation, a suprapubic or short midline incision is advised.

33/37 = 89.2%

In all cases, a wound protector should be used to reduce the potential risk of wound infection and tumour cell implantation.

35/37 = 94.6%

### Outcome analysis

#### Outcome research

Several publications from expert centres have shown promising results and have supported the dissemination of TaTME. The consensus panel encourages the prospective monitoring of perioperative clinical outcomes, histopathology results, oncological outcomes, and function/quality of life.

37/37 = 100%

Clinical outcomes of interest include operative time, one-team or two-team procedure, conversion to laparoscopy, conversion to open surgery, 30-day post-operative morbidity taking advantage of a validated grading system (e.g. the Dindo-Clavien classification [21]), length of hospital stay, and readmission. Anastomotic leaks should be graded according to the international grading system (A–C) [22]. Whether a case has been proctored should also be recorded.

37/37 = 100%

Oncologic outcomes of interest include quantification of resection margins (proximal, distal, and circumferential resection margins), Quirke/Mercury TME quality grading [23], TNM staging [24], including the number of lymph nodes retrieved, and both neoadjuvant and adjuvant treatment. Recurrence rates shall be monitored prospectively as well.

37/37 = 100%

Functional outcomes of interest include generic and colorectal cancer specific assessment of quality of life (e.g. EORTC QLQ-C30 & CR-29 [25]), urinary function (e.g. IPSS [26]), gender specific sexual function (e.g. IIEF-5 [27]/ FSFI-6 [28]), bowel function (e.g. LARS [29], Vaizey [30]), and health utility (e.g. EQ-5D [31]).

36/37 = 97.3%

Lastly, econometric analysis may be of interest, including assessment of health utilities (e.g. EQ-5D [31]), procedure and hospital costs, and hospital reimbursement.

37/37 = 100%

#### Registries and clinical trials

The consensus panel strongly advises participation in the international TaTME registry [3] (http://www.TaTME.surgery) and in the randomised controlled trials COLOR III [4] and GRECCAR 11 [5] (TaTME vs laparoscopic TME).

37/37 = 100%

### MRI staging and prognosis information

*Gina Brown, Consultant Radiologist*

Multidisciplinary review of MRI rectal cancer findings leads to improved outcomes [32]. Thus, MRI is the investigation of choice for local staging of rectal cancer. It shows the extension of the tumor through the rectal wall and to the mesorectal fascia, and the involvement of local nodes and vessels [33, 34]. Further, MRI enables an objective assessment of the tumour with respect to the sphincter and the anal verge, guiding management decisions regarding potential sphincter preservation [35]. Understanding the relationship of the tumour to the distal TME plane at or just above the puborectalis sling prevents inadvertent surgical perforation and dissemination of tumour during distal TME dissection. Also, it allows selective use of extralevator abdominoperinal excision for those tumours where the invasive border lies within 1 mm of the intersphincteric plane, levator muscle, or lower prostate [36, 37]. Last, MRI assessment reveals the area or quadrant of maximal tumour at risk of margin involvement [36].

Other prognostic and predictive factors that are assessed include depth of extramural spread (mrT substage) [38, 39] and the presence or absence of tumour signal into extramural veins [40–42]. High resolution MRI technique enables characterisation of nodes based on breach of the nodal capsule and/or replacement of nodal tissue by tumour resulting in border irregularity and mixed signal intensity respectively. Size criteria should not be used [34, 43]. Compared with the assessment of mrCRM (TME plane), millimetre assessment of the extramural depth of spread, and MRI assessment of venous invasion, MRI assessment of nodes do not hold prognostic significance [44]. Reporting standards for MRI staging are shown in Table 1.

**Table 1:** Reporting standards for MRI staging

MRI reporting standards
Site of tumour—upper/mid/lower third
Height from puborectalis sling and anal verge and craniocaudal length
For tumours arising at or within 2 cm above the level of the puborectalis sling—assessment of the safety of the total mesorectal excision (TME) surgical plane
Relationship to important landmarks, e.g., peritoneal reflection/seminal vesicles
Morphology: e.g. annular/semi-annular/mucinous Infiltrating border—smooth or nodular infiltration
Presence or absence of extramural venous invasion Presence or abscence of vascular mediate tumour deposits (N1c)
Maximum depth of extramural spread
Presence or absence of malignant lymph nodes (smooth border/uniform signal = benign irrespective of size)
Minimum distance to mesorectal fascia or intersphincteric plane > 1 mm = mrCRM clear
In the final assessment, the TNM stage and an assessment of potential resection margin involvement/safety of the TME plane (classified as potentially involved if tumour < 1 mm to the mesorectal fascia/ intersphincteric plane) should be made

### A word of caution by the pathologist

*Philip Quirke, Consultant Pathologist*

As any new technique, TaTME needs rigorous evaluation and its safety needs to be proven. Pathology is helpful in evaluating its effectiveness and optimizing surgical technique. Major changes in local recurrence and survival in rectal cancer have been achieved by considering the anatomy of the rectum, careful selection of the appropriate planes on MRI, assessing the surgeon’s ability to deliver the appropriate anatomical package and quality assurance by the pathologist through describing the involved margin rate [45] and the quality of the surgery [23, 46, 47].

The anatomy is difficult as posteriorly there are complex changes in the angulation of the mesorectal fascial plane and the mesorectum is initially a thin fatty layer. Anteriorly there is very little mesorectum and the anterior surgical margin is in juxtaposition to the urethra. In the male important nerves run at the height of the prostate, so surgery needs to be very precise. It is essential that the surgeon finds and develops the correct plane around the tumour and stays within it. Since the height of the tumour is an important factor with lower tumours generating worse surgical planes [47] there may well be a place for excellent TaTME but this needs to be proven. The degree of increased operative difficulty caused by preoperative therapy also needs to be determined.

Auditing of key pathology features safeguards the quality of the surgical planes, especially anteriorly in the low rectum, and the frequency of CRM positivity. Photography of the anterior and posterior surfaces of all TaTMEs is essential to alert surgeons to suboptimal planes and ensure their correction in future cases. Photography allows for external audit and evidence rather than opinion based audit. Early, self-declared registry information looks optimistic [48] but this is no substitute for proper cohort studies with central pathology evaluation and MRI stratified randomised trials.

### Multimodal therapy and role of the multidisciplinary team

*Ulrich Güller, Consultant Medical Oncologist*

Multimodal therapy and multidisciplinary tumour board discussions resulted in a quantum leap regarding outcomes of rectal cancer patients. However, major challenges still lay ahead: First, while local relapses have become a rare phenomenon in patients undergoing neoadjuvant chemo-radiotherapy and proper TME, distant metastases remain an unsettling problem. Hence, further efforts must focus on improving systemic treatment. Second, not all patients need the trimodal therapy including radiation, surgery, and chemotherapy. Currently, randomised trials are evaluating whether patients with good response to neoadjuvant chemotherapy can do without additional radiation (PROSPECT Trial, Alliance) [49]. Moreover, the use and time point of chemotherapy are further evaluated as it is hypothesized that systemic treatment is used too late in the treatment sequence leading to a higher risk of distant relapse. Contemporary randomised controlled trials now evaluate total neoadjuvant treatment (e.g. Rapido Trial [50]), in which patients receive chemotherapy in a neoadjuvant setting. Finally, not all patients must undergo surgery. As pioneered by Habr-Gama [51], a relevant fraction of patients having a complete clinical response after neoadjuvant therapy can be followed without immediate operation, with good outcomes, including improved quality of life and decreased morbidity, as the recent presentation at ASCO GI 2017 of the International Watch and Wait Database (IWWD) for Rectal Cancer confirms [52]. To further advance care and knowledge in treating rectal cancer patients, it is of cardinal importance to nurture an ongoing collaboration with all actively involved disciplines including surgery, radiation oncology, gastroenterology, pathology, radiology and medical oncology.

## Discussion

The primary objective of this interdisciplinary consensus statement was to provide guidance to those embarking on TaTME. Secondary, it was aimed to highlight the challenges, benefits, and distinctive dangers of this technique, and to promote prospective outcomes analysis.

Seven different aspects were analysed including: patient selection and indication, perioperative management, patient positioning and operating room set up, devices and instruments, pelvic anatomy, TaTME training, and outcomes analysis. Globally, the statements achieved more than 80% approval for most of these items, which were graded as strong recommendations. However, it is acknowledged that there is a current lack of high level evidence in support of this recommendation, which is based only on expert opinion. On the other hand, the panel of experts found a large agreement on all the different questions.

As for recommendations, the GRADE guidelines [6] state: ‘Strong recommendations indicate that the panel is confident that the desirable effects of adherence to a recommendation outweigh the undesirable effects. Weak recommendations indicate that the desirable effects of adherence to a recommendation probably outweigh the undesirable effects, but the panel is less confident’. Recommendations were based not only on the quality of evidence but also on the balance between wanted and unwanted effects, and on values and preferences [53]. The latter implies that, in some instances, strong recommendations may be reached from low-quality data and *vice versa*.

The introduction of a new technique must occur in a safe and controlled manner to protect both the patient and the surgeon. The expert panel agreed that the earlier stages of the learning curve are best overcome by initially selecting easier cases, although no agreement was reached on gender selection with 60% of the panel favouring the broader pelvis of female patients to begin with TaTME. However, the panel acknowledges that the greater the BMI and/or more unfavourable the hip-waist ratio the more TaTME helps to overcome the challenges of an oncologic low pelvic dissection. This is particularly true in male patients with low rectal cancer.

The adoption of TaTME has seen an exponential growth worldwide. The largest cohort to date includes recently published results from the International TaTME registry, suggesting an oncologically safe and effective technique with acceptable short-term clinical outcomes [48]. However, surgeons did experience intra-operative equipment and technical difficulties in up to 40% of cases, with incorrect plane dissection, pelvic bleeding, unstable pneumopelvis and, more worryingly, visceral injuries such as urethral division. Indeed, one of the most dreaded specific complication of TaTME is the injury of the urethra during initial anterior dissection. The TaTME surgeon has to recognize new landmarks in dissection and think in different planes. The prostate may initially appear as a vertical wall in front of the dissection and inadvertently be dissected en bloc with the anterior mesorectum or ‘dug’ into, causing urethral injury. Removing the platform to palpate and help define the anatomy of the prostate/urethra or vagina is a key step in case of doubt. Similarly, for very low tumours where dissection is initiated in an intersphincteric plane the platform may be inserted once a classical transanal dissection using a conventional retractor has clarified the surgical/oncological plane. Later during the high lateral dissection, the autonomic nerves, ureters, and pelvic vessels are at risk. In case of major bleeding, tamponade by a transanally inserted gauze and positioning the patient in reverse Trendelenburg help control and repair vascular injury, which may be completed laparoscopically.

This consensus strongly recommended proper training, including participation in dedicated courses and proctoring of the first cases before embarking on independent practice of TaTME. Guidance from surgeons experienced in TaTME help new adopters of the technique avoid mistakes made in the past and progress at an efficient pace with more appropriate and specialised equipment becoming widely available. To start a TaTME practice, a minimal annual volume of 10 complete TME dissections for cancer was agreed on. This figure, although low, was felt the least to achieve but many voiced concerns that more may be required to obtain best possible results. A minimum learning curve of 20 cases performed within about 2 years was felt reasonable, while it was recognized that individual variability may influence the length and steepness of the learning curve. Experience of the surgeon and his team should be considered when reporting and appraising outcomes. Importantly, participation to an international registry and/or clinical trial is encouraged to share experience and benchmark one’s practice with other surgeons and institutions.

Whilst this consensus did focus on malignant pathologies requiring TME, this new approach may be applied for benign conditions as well, although this emerging indication was beyond the scope of the present consensus. Several reports have shown benefits of a transanal approach beyond cancer [54–58], with 11.9% of the cases reported in the TaTME registry [48] addressing benign conditions. Most benign procedures were an intersphincteric amputation or a proctectomy with ileal-pouch-anal reconstruction for inflammatory bowel disease. A transanal approach facilitates proctectomy, especially in obese patients with narrow pelvis. Also, it allows an exact transection of the rectum at the top of the anal canal, leaving no rectal mucosa behind, and avoids multiple stapler firings and cross-stapling. Further benign indications include complex fistulae [59, 60], anastomotic complications (stenosis or leakage) [61–63], completion proctectomy [64–66], deep pelvic endometriosis [67], and reversal of Hartmann [68].

## Conclusion

A broad international consensus statement is presented herein, which provides a basis for optimal clinical practice. This multidisciplinary consensus statement achieved more than 80% approval and can thus be graded as strong recommendation, yet acknowledging the current lack of high level evidence. It provides the best possible guidance to safe implementation of Transanal Total Mesorectal Excision.
